# Postoperative Analgesia With Modified Thoracoabdominal Nerve Block Through Perichondrial Approach in Neonatal and Infantile Abdominal Surgery

**DOI:** 10.7759/cureus.65219

**Published:** 2024-07-23

**Authors:** Hirofumi Nakahari, Tomoyo Takahashi, Hayato Miki, Akihiko Yamaguchi

**Affiliations:** 1 Anesthesiology, St. Luke's Hospital, Tokyo, JPN; 2 Anesthesiology, St. Luke’s Hospital, Tokyo, JPN

**Keywords:** infants, ultrasound-guided regional anesthesia, pediatric regional anesthesia, pediatric pharmacology, upper abdominal surgery, analgesia, local anesthetic, neonates

## Abstract

Modified thoracoabdominal nerve block through the perichondrial approach (M-TAPA) is a novel strategy for peripheral nerve block in the abdomen. Its usefulness has been highlighted in adults, but no literature is currently available regarding its efficacy in infants. This report describes the cases of a one-day-old neonate in open abdominal surgery and a one-month-old infant in laparoscopic surgery who received M-TAPA. The postoperative condition of the infants was assessed through a neonate pain scale and the Face, Legs, Activity, Cry, and Consolability behavioral scale, respectively; both scales remained at 0 until discharge. Despite the need for special attention, M-TAPA may provide effective analgesia in neonatal and infant abdominal surgery in addition to adult cases, and its indications should be considered.

## Introduction

Appropriate pain management is important for early postoperative recovery of all cohorts. Recently, multimodal analgesia combining regional anesthesia with non-opioid analgesics has become a common strategy to prevent excessive postoperative opioid use. Specifically, in infants younger than six months of age with immature liver function and reduced clearance of opioids, sparing opioids postoperatively with multimodal analgesia combining regional anesthesia may prevent the incidence of opioid-related complications, such as respiratory depression, inhibition of intestinal motility, and opioid tolerance, and contribute to improving both short-term and long-term patient outcomes. Initiating intestinal nutrition as soon as possible, especially during infancy, is extremely important. Recently, nutritional management during the early postnatal period in infants has been reported to have a significant impact on the development of the central nervous system [1]. Long-term effects on neurocognitive development and risk factors for non-communicable disease in adulthood have been suggested. Considering these aspects, appropriate postoperative pain management and prevention of opioid overuse are critical to promote intestinal nutrition by maintaining normal function of the gastrointestinal tract [1].

Ultrasound-guided peripheral nerve block techniques are commonly performed, and the observed safety features have led many institutions to adopt the practice as an alternative to spinal canal anesthesia. Nowadays, several approaches have been reported for peripheral nerve blocks in the abdomen. In 2019, Tulgar et al. described thoracoabdominal nerve block through the perichondrial approach (TAPA) and modified TAPA (M-TAPA) as a novel approach [2,3]. TAPA consists of the injection of local anesthetics into two layers: the first injection is between the internal oblique and transversus abdominis muscles located under the ninth and 10th costal cartilages, and the second is between the external oblique and the costal cartilage at the level between the ninth and 10th costal cartilages [2]. M-TAPA includes only the first injection [3]; it provides anesthesia to both the anterior and lateral cutaneous branches of the thoracoabdominal nerve and shows potent analgesic effects over wide areas of the abdomen [3]. The modified approach has been used in many abdominal surgeries in adults, and various case reports and clinical studies have confirmed its effectiveness [4]. To the best of our knowledge, reports of pediatric cases are limited regarding M-TAPA [5], and case reports or clinical studies on M-TAPA application, especially in the neonatal or infantile period, are lacking.

This report describes two cases of the application of M-TAPA with successful analgesia in a neonate and an infant who underwent abdominal surgery.

## Case presentation

Case 1

A one-month-old boy (height, 56.4 cm; weight, 5.1 kg) presented with projectile vomiting and was diagnosed with hypertrophic pyloric stenosis. Preoperative laboratory examination revealed no electrolyte abnormalities or evidence of background disease impending surgery. A laparoscopic pyloromyotomy was scheduled on the same day. 

**Figure 1:**
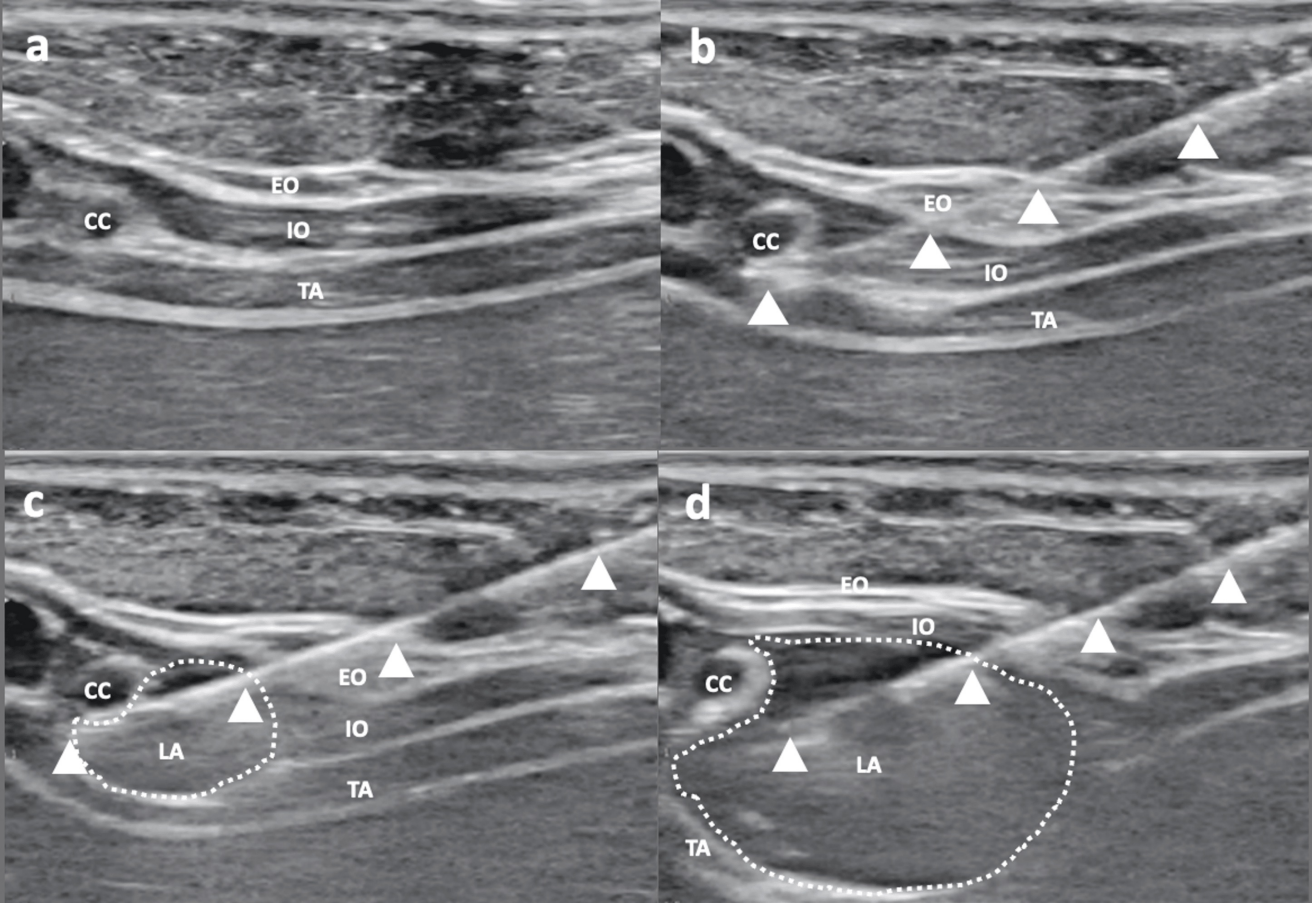
Ultrasound views during M-TAPA (a) Ultrasound image of the perichondral area before blocking. (b) Sonographic view of the block needle before injection. (c) Sonographic view of the block needle and injection point at the lower aspect of the chondrium. (d) Ultrasound image after the completion of M-TAPA. CC, costal cartilage; EO, external oblique muscle; IO, internal oblique muscle; TA, transversus abdominis muscle; LA, local anesthetic; M-TAPA, modified thoracoabdominal nerve block through the perichondrial approach

No premedication was administered. An intravenous line was secured prior to the induction of anesthesia. An oral gastric tube was inserted before induction of anesthesia to prevent aspiration, and gastric contents were adequately removed. The patient’s vital signs, such as electrocardiogram (ECG), pulse rate, oxygen saturation, end-tidal carbon dioxide concentration, blood pressure, and body temperature, were continuously monitored during general anesthesia. Following preoxygenation, modified rapid sequence induction (M-RSI) was initiated with propofol 2.0 mg/kg, fentanyl 2.0 μg/kg, and rocuronium 1.2 mg/kg. The patient’s airway was secured with a 3.0 mm cuffed tracheal tube. General anesthesia was maintained by using sevoflurane (end-tidal concentration, 0.5-1.0%) and remifentanil infusion (0.5-1.0 μg/kg/min) with adjusting the patient’s vital signs. Rocuronium was added intermittently during surgery. Fentanyl was not administered additionally after induction, considering postoperative extubation. Surgery proceeded with placing three laparoscopic ports in the umbilicus and right and left upper abdomen. At the end of surgery, acetaminophen 40 mg was administered intravenously. Before emergence from general anesthesia, ultrasound-guided M-TAPA was performed bilaterally using a nerve block needle (22 G × 50 mm; Stimuplex Ultra 360; B. Braun, Tokyo, Japan) (Figure 1). Levobupivacaine 1.5 mg/kg was diluted with 10 mL of saline, and 5.0 mL was injected into each side after a negative aspiration test. Following the completion of M-TAPA, sugammadex was administered, and the tracheal tube was extubated after confirming adequate emergence and spontaneous breathing. The patient was transferred to the general ward. 

The postoperative condition was assessed using the Face, Legs, Activity, Cry, and Consolability (FLACC) scale [6]. FLACC scale is recommended for pain assessment in infants and toddlers who have difficulty communicating verbally and assesses five behaviors, which are facial expression, leg movement, activity, crying status, and consolability. Each behavior is scored from 0 to 2, with the total score ranging from 0 (no pain) to 10 (most pain). FLACC scores greater than 6 indicate distress and require treatment [6]. During the hospital stay, the score remained at 0 without additional analgesics. The patient was discharged on postoperative day 3 without any complications.

Case 2

A one-day-old boy (height, 52.0 cm; weight, 3834 g) presented with an anorectal malformation at birth, and after further evaluation, a diagnosis of anal atresia was made. Jejunoileal atresia was also suspected, and a nasogastric tube was inserted to decompress the bowel and prevent aspiration. No other congenital diseases were identified, and colostomy was scheduled on day 1.

**Figure 2:**
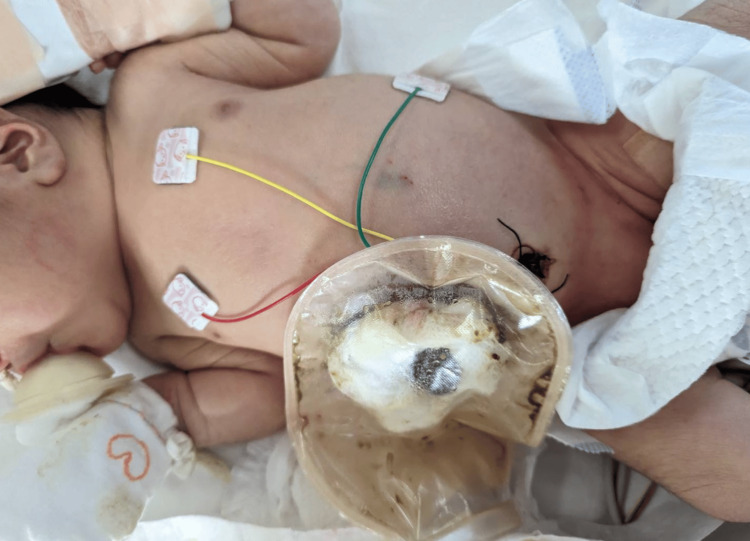
Photograph from the neonatal intensive care unit after surgery The surgical site was on the right upper abdomen, and a colostomy was placed in the same site.

No premedication was administered, and an intravenous line was secured before anesthesia induction. The patient’s vital signs were monitored during the general anesthesia. Following preoxygenation, M-RSI was initiated with propofol 2.0 mg/kg, remifentanil 2.0 μg/kg, and rocuronium 1.2 mg/kg. The airway was secured with a 3.0 mm cuffed tracheal tube. General anesthesia was maintained using sevoflurane (end-tidal concentration, 0.4-1.5%) and remifentanil infusion (0.4-1.0 μg/kg/min) while monitoring the patient’s vital signs. Rocuronium was added intermittently, and fentanyl was not administered throughout the surgery since the patient was planned to extubate in the operating room. Ultrasound-guided M-TAPA was performed bilaterally before the surgical incision. Levobupivacaine 1.5 mg/kg was diluted with 10 mL of saline, and 5.0 mL was injected into each side after confirming the aspiration test. The surgical incision site was in the right upper abdomen, and a colostomy was created at the same site (Figure 2). Acetaminophen 30 mg was administered intravenously before the end of surgery. Postoperatively, sugammadex was administered, and the tracheal tube was extubated after confirming adequate emergence and spontaneous breathing. The patient was transferred to the neonatal intensive care unit (NICU). 

The postoperative condition was assessed using the Neonatal Infant Pain Scale (NIPS) [6]. NIPS is adopted in our NICU, and the assessment parameters include facial expression, cry, breathing pattern, arm and leg movement, and arousal state. Each of these parameters is scored on a scale from 0 to 2, with the total score ranging from 0 (no pain) to 7 (most pain). Treatment might be necessary for neonates with a score of 4 or more [6]. From neonates, regardless of preterm or full term, to infants around one month of age are considered to be the recommended period of the scale’s application. Except for non-pharmacological pain management (non-nutritive sucking, swaddling, and facilitated tucking) routinely performed in our NICU, NIPS remained 0 without extra-analgesics during the NICU stay. The patient was discharged on postoperative day 21 without any complications.

## Discussion

We describe two cases of infants who underwent abdominal surgery with successful postoperative analgesia using M-TAPA. Possible options for postoperative analgesia in the presenting case included thoracic epidural anesthesia, caudal block, and peripheral nerve block. Thoracic epidural anesthesia provides effective analgesia in adults; however, complications such as accidental dural puncture and severe neurological damage were reported, especially more often in neonates and infants. Therefore, many reports recommend epidural anesthesia for children be performed by an experienced pediatric anesthesiologist [7]. Caudal block is performed in many institutions, even in the neonatal period, although many local anesthetics are required to extend the analgesic range to the upper abdomen. Furthermore, the duration of analgesia from a single injection of epidural anesthesia in infancy is reported to be approximately 90-120 minutes, requiring the insertion of a catheter for postoperative analgesia [7]. Especially when an epidural catheter is inserted by the caudal approach and placed at the thoracic level, the possibility of intravascular or subarachnoid entry increases with the increased length of catheter insertion. In addition, complications such as nerve root damage and catheter dislocation may increase in frequency. Considering these potential risks, we decided on peripheral nerve block as the first option for postoperative analgesia. M-TAPA is a novel peripheral nerve block with the potential to target both the anterior and lateral cutaneous branch regions of the thoracoabdominal nerve with a single injection [3,4]. Since the surgical sites of the two presented cases both partially exceeded the midline, we decided to select M-TAPA to cover the anterior and lateral cutaneous branch area. 

Perioperative management during infancy requires attention to long-term neurocognitive and neurobehavioral outcomes of infants. Preclinical studies have revealed that the use of gamma-aminobutyric acid (GABA) receptor agonists and N-methyl-D-aspartate (NMDA) receptor antagonists, such as inhalational anesthetics, propofol, benzodiazepines, and ketamine, cause neuronal apoptosis in a volume-dependent manner [8]. However, there are still inconclusive results regarding the effects of exposure to such anesthetics in human infants on later development [8]. Opioids and dexmedetomidine, in contrast, have less evidence of causing neurodegeneration [9]. Currently, ongoing research is designed to evaluate new anesthetic regimens based on these anesthetics in neonates and infants [9]. Based on the available evidence, clinicians should adopt the strategy of using anesthetics with less impact on neuronal apoptosis, such as opioids, and try to reduce the use of anesthetics that may cause volume-dependent neuronal apoptosis. According to this concept, opioid-based anesthesia using remifentanil was applied to maintain general anesthesia in the presented two cases, and the concentration of inhalational anesthetics was adjusted to the minimum necessary while assessing the vital signs. Since remifentanil has the highest clearance and largest distribution volume in infancy [10], approximately double the dosage is required to achieve the same effect site concentration as in adults. Except for the dosing, the efficacy of remifentanil is similar in adults. Since it is an ultra-short-acting opioid, even in neonates, no effect on postoperative respiratory depression has been reported [10]. Remifentanil contributed greatly to facilitating the management of both our patients being planned to extubate in the operating room.

In addition to the effects of anesthetics on the developing brain, the long-term effects of pain during infancy require special consideration. The details regarding pain pathways in infancy, including the degree and manner of perception and impact on later development, remain unknown. Over the past few decades, various studies have investigated pain in infancy, and the approach to infantile pain has changed. Previously, infants were considered to experience less pain due to immature neurological development. Recent reports suggest that infancy is associated with a greater function of cutaneous receptor fields and immature descending inhibitory pathways, resulting in augmented pain signals and stress responses compared to adults [11]. Additionally, neonates exposed to persistent and significant pain in perioperative or perinatal periods at NICU respond stronger to nociceptive stimuli in later life than those who do not experience such pain [12]. An association between pain experienced in childhood and developmental problems or behaviors has also been suggested [13]. Appropriate strategies to control pain are necessary to prevent these issues. Regional anesthesia could make a significant contribution in this regard.

Administration of regional anesthesia should be considered carefully in pediatric patients due to their unique anatomical and physiological characteristics. First, the blood concentration of α1-acid glycoprotein, a plasma protein that binds to local anesthetics, is low (0.2-0.3 g/L) and does not reach adult levels (0.7-1.0 g/L) until one year of age [14]. This increases the blood concentration of free local anesthetics (not combined with α1-acid glycoprotein), leading to local anesthetic systemic toxicity (LAST) more easily than in adults. Therefore, the maximum dose of local anesthetics must be reduced in this age group. Second, nerve fiber myelination is incomplete, especially in infants. Myelination of both the peripheral and central nervous system begins during the fetal period and is not largely completed by 12-24 months of age [15]. A major pharmacological consequence of this condition is the facilitation of the penetration of local anesthetics into nerve fibers, resulting in easier nerve blockade while simultaneously indicating potential neurotoxicity. The minimum effective local anesthetic concentration (MLAC) in infancy for various nerve blocks remains unclear due to insufficient evidence. MLAC decreases with age, with some reports indicating by half or less [16].

A previous article described LAST after performing erector spinae plane block in a neonate [17]. According to this report, a neonate weighing 4.0 kg developed LAST after receiving 2.0 mL of 0.2% levobupivacaine (1.0 mg/kg), much less than the dose used in our practice. Possible explanations for the absence of LAST events in our cases are the increased blood concentration of α1-acid glycoprotein due to inflammation and stress associated with the surgical incision [18], which reduced the fraction of free levobupivacaine in the bloodstream. Another considerable contribution is the concentration (approximately 0.05%) of levobupivacaine we injected, which may have helped prevent a rapid increase in the blood concentration of free levobupivacaine. Based on the unique characteristics of infants described above, it is important to consider using lower concentrations of local anesthetics to minimize the neurotoxicity of local anesthetics and prevent LAST. Clarification of MLAC regarding the neural structure of infants may provide criteria for the use of local anesthetics in infants; further research is needed.

Accurate pain assessment is especially challenging in neonates and infants, as they cannot verbalize their pain experience. Although several observational scales have been developed and validated, all have limitations in terms of accuracy, as they rely on individual discretion and judgment in many assessment categories, which makes it difficult to reach the gold standard [19]. Recently, the Neonatal Parasympathetic Evaluation (MDoloris Medical Systems, Loos, France) monitor was developed to overcome limitations in the pain assessment scale and has been validated in children younger than two years [20]. The technology was developed based on the neurophysiological response to pain, specifically on the premise that a painful stimulus suppresses the parasympathetic nervous system. The monitor analyzes high-frequency (0.15-0.40 Hz) heart rate variability as a measure of parasympathetic tone. The technique is non-invasive and presents a continuous pain index by analyzing electrocardiogram signals through a connection to a cardiac monitor [20]. Future development of such tools that can objectively assess the degree of pain will be interesting. These tools could help develop and generalize pediatric regional anesthesia.

## Conclusions

The active application of M-TAPA in infant abdominal surgery could be a reasonable option for postoperative pain management. In our opinion, as long as anesthesiologists understand the neurophysiological characteristics of infants and use this knowledge appropriately, regional anesthesia could be safely and effectively performed in infants as with adults. Using an appropriate pain assessment scale and providing multimodal analgesia, including regional anesthesia, may help prevent the long-term effects of pain and improve later life outcomes in children. We anesthesiologists have a responsibility to protect the future of children who unfortunately require surgery in infancy, and every effort should be made to fulfill this responsibility.
